# Advancing Health Equity for American Indian and Alaska Native People Through Inclusion in Clinical Trials: Anti-SARS-CoV-2 Monoclonal Antibody Treatment and COVID-19 Outcomes Among Ambulatory Cherokee Nation Health Services Patients

**DOI:** 10.1089/heq.2024.0185

**Published:** 2025-04-21

**Authors:** Jorge Mera, Whitney Essex, Elizabeth Menstell Coyle, Ashley Comiford, Molly A. Feder

**Affiliations:** ^1^ Department of Infectious Diseases, Cherokee Nation Health Services, Cherokee Nation Outpatient Health Center, Tahlequah, Oklahoma, USA.; ^2^ Cardea Services, Seattle, Washington, USA.; ^3^ Sea Glass Consulting, Seattle, Washington, USA.

**Keywords:** American Indian/Alaska Native, health equity, medical research, monoclonal antibody

## Abstract

**Background:**

Racial/ethnic minority groups are underrepresented in clinical trials with American Indian and Alaska Native (AI/AN) people having the lowest representation. This article aims to contribute to the literature to address that gap by sharing the results of the use of anti-SARS-CoV-2 monoclonal antibodies among AI/AN people at risk for severe COVID-19.

**Methods:**

This retrospective cohort study assessed data from ambulatory AI/AN patients enrolled in Cherokee Nation Health Services in Northeastern Oklahoma, who had a positive test for SARS-CoV-2, high risk for progression, and were offered anti-SARS-CoV-2 monoclonal antibody treatment active against the circulating SARS-CoV-2 strain from December 1, 2020, to April 16, 2021. The outcomes of interest were all-cause and COVID-19-related emergency department visits, hospitalizations, intensive care admissions, and deaths within 28 days of being offered treatment.

**Results:**

Among 1,447 participants, 813 (56.2%) were treated and 634 (43.8%) were not. When adjusted for potential confounders, there was a significant difference in the odds of treated versus untreated patients experiencing a COVID-19-related emergency department visit (OR, 0.42; 95% CI, 0.27–0.63) and hospitalization (OR, 0.10; 95% CI, 0.03–0.31).

**Discussion:**

Anti-SARS-CoV-2 monoclonal antibody treatment was associated with lower odds of COVID-19-related emergency department visits and hospitalization among high-risk AI/AN patients.

**Health Equity Implications:**

To advance health equity, it is critical to have representation of AI/AN in clinical trials and other research. This project is an example of how community partnerships with AI/AN health systems can strengthen the evidence for new and emerging treatments, address past harm, and advance equity.

## Introduction

Racial/ethnic minority groups are underrepresented in clinical trials with AI/AN people having the lowest representation.^1,2^ Advancing health equity for AI/AN people requires an understanding of the complex and intersecting historical, systemic, structural, and institutional factors that influence the health and well-being of Indigenous people. Factors include the transgenerational effects of colonialism, racism, and trauma and significant barriers to accessing health care, based, in part, on chronic underfunding of the Indian Health Service and other Tribal and Urban Indian health sites, geographical isolation, and economical barriers.^3–5^ This article aims to contribute to the literature to address that gap by sharing the results of the use of anti-SARS-CoV-2 monoclonal antibodies among AI/AN people at risk for severe COVID-19 in the Cherokee Nation.

Among racial and ethnic groups, COVID-19 is disproportionately impacting AI/AN people: the cumulative incidence of COVID-19 in AI/AN people is 3.5 times that of white people^6^ and the highest rate of mortality due to COVID-19 compared to other racial and ethnic groups.^7^ As of June 1, 2024, Cherokee Nation had 60,153 positive cases of COVID-19, as noted in Cherokee Nation Health Services (CNHS) medical records (unpublished data). Although widespread distribution of COVID-19 vaccinations has reduced the number of people experiencing hospitalization and death,^8–10^ there is still a need to improve COVID-19 treatment and health outcomes among AI/AN people.

In November 2020, the U.S. Food and Drug Administration (FDA) approved the first monoclonal antibody treatment for COVID-19, under an emergency use authorization (EUA).^11^ Since then, several anti-SARS-CoV-2 monoclonal antibody products have received EUA. AI/AN people have been underrepresented in monoclonal antibody treatment research, including initial clinical trials by Regeneron Pharmaceuticals and Eli Lilly and Company in which a total of 11 (0.3%)^12^ and 5 (0.4%)^13,14^ AI/AN people participated, respectively. Only one study, a quality improvement study by Close et al., has focused on AI/AN people.^15^ Consequent to the underrepresentation of racial and ethnic minorities in research, there is limited understanding of the efficacy of COVID-19 treatment among AI/AN people, despite disproportionate morbidity and mortality.

The Cherokee Nation is the largest federally recognized tribal nation in the United States, spanning 14 counties in Oklahoma, and includes more than 450,000 registered Cherokee citizens.^16^ CNHS, Cherokee Nation’s network of health centers, is the largest tribally operated health system in the United States.^17^ In December 2020, Indian Health Services began providing bamlanivimab, the anti-SARS-CoV-2 monoclonal antibody effective against the COVID-19 variant circulating at the time (alpha variant, B.1.1.7^18^) to CNHS for use among patients with COVID-19 under an FDA EUA. Due to the high volume of patients meeting the EUA criteria and limited availability of treatment, the CNHS had to prioritize treatments based on patient risk factors for progression to severe disease. Selection of risk factors was based on those most frequently encountered in patients who had been hospitalized at that point in time in the CNHS and included being ≥65 years, having a body mass index (BMI) ≥35, and having diabetes, as recorded in CNHS medical records (unpublished data).

The primary aim of this study was to assess if receipt of a historical treatment for COVID, anti-SARS-CoV-2 monoclonal antibody, was associated with decreased rates of emergency department visits, hospitalizations, intensive care unit admissions, and deaths among AI/AN patients with mild to moderate COVID-19 and high risk of disease progression. Through sharing this knowledge with the scientific community, we hope to contribute to the efforts of those who advocate for the inclusion of AI/AN patients in clinical trials and other medical research to support decision-making around AI/AN people’s health and well-being.

## Methods

The Cherokee Nation Institutional Review Board approved this retrospective cohort study. This study followed the Strengthening the Reporting of Observational Studies in Epidemiology reporting guideline.^19^

### Setting

The study was conducted through CNHS which operates in Northeastern Oklahoma across 11 health care facilities, including one hospital, nine outpatient health facilities, and one employee health center.^17^

### Participants

Participants were included in this study if they were contacted to receive treatment from December 1, 2020, through April 16, 2021. Participants included AI/AN people eligible for health care through CNHS, who had a positive polymerase chain reaction or antigen test for SARS-CoV-2. To receive health care at CNHS, individuals must provide proof of enrollment as a member or citizen of a federally recognized tribe. Patients who met the CNHS eligibility criteria for monoclonal antibody treatment were contacted by the nurse monitoring team. Eligibility criteria included having COVID-19 symptoms within the last 10 days and having one of the following: ≥65 years, BMI over 35, or diabetes. Patients who required hospitalization on the day of evaluation for monoclonal antibody treatment were not given treatment. Criteria for hospitalization were oxygen saturation (SpO_2_) <94%, respiratory rate of >30, or hypotension BP <90/60.

### Data sources

The data used for this study were de-identified electronic medical record (EMR) data, collected by CNHS clinical staff as part of standard patient care procedures. CNHS’s EMR system includes data from all patients who have had at least one encounter with CNHS.

### Exposure and outcome variables

The primary exposure of interest was receipt of anti-SARS-CoV-2 monoclonal antibody treatment (bamlanivimab) versus standard care. Standard care for COVID-19 at the time of data collection for this study included symptom treatment, infection control measures, and follow-up by a nurse monitoring team.

The primary outcomes of interest included the occurrence of all-cause and COVID-19-related hospitalization, emergency department visits, intensive care unit admission, and death. Outcomes had to have been recorded in the patient’s EMR within 28 days of the phone call by the nurse monitoring team to be included in this study. If individuals did not have an outcome recorded within 28 days, they were considered as not having the outcome. All-cause versus COVID-19-related outcomes were determined by chart review by an infectious disease provider. Providers determined that the emergency department admission and hospitalization were COVID-related when the primary diagnosis was directly related to COVID-19, such as respiratory distress or pneumonia. All-cause emergency department admission and hospitalization was defined as any admission, regardless of the underlying cause. This includes any medical, surgical, or other conditions that necessitate emergency department admission or hospitalization, such as trauma, chronic disease exacerbations, or elective procedures, including cases where a patient tested positive for COVID-19.

Covariates for this study included age, sex (male/female), calculated BMI, and presence of the following preexisting conditions: diabetes, heart disease/hypertension, chronic kidney disease, and chronic lung disease. Covariates were confirmed with patients when screened by the nurse monitoring team. All covariates were selected for inclusion in statistical models *a priori* as they have been shown to be associated with COVID-19-related outcomes in prior studies.^20–24^

COVID-19 vaccination status was considered as a potential covariate for this study. Upon review of data, there were only two participants who were fully vaccinated for COVID-19 prior to this study. For this reason, COVID-19 vaccination was excluded as a covariate.

We computed one variable for this study. This variable was the days between COVID-19 symptom onset and treatment. To compute this variable, we calculated the number of days between patient-reported COVID-19 symptom onset date and date of receipt of anti-SARS-CoV-2 monoclonal antibody treatment.

### Statistical analysis

Differences in characteristics between treated and untreated patients were initially assessed using chi-square and Fisher’s exact tests. Quantitative patient data were then analyzed using binary and multivariable logistic regressions, including all covariates, to determine the crude and adjusted odds of the outcomes of interest occurring among patients who did and did not receive anti-SARS-CoV-2 monoclonal antibody treatment after receiving a positive COVID-19 test result.

Among the participants in the exposure group, the average number of days between COVID-19 symptom onset and treatment was assessed using counts, proportions, means, standard deviations, chi-square tests, and Fisher’s exact tests.

All quantitative analyses were performed using SPSS version 19 and R 4.1.2 statistical analysis software.

## Results

### Study sample

**FIG. 1.**
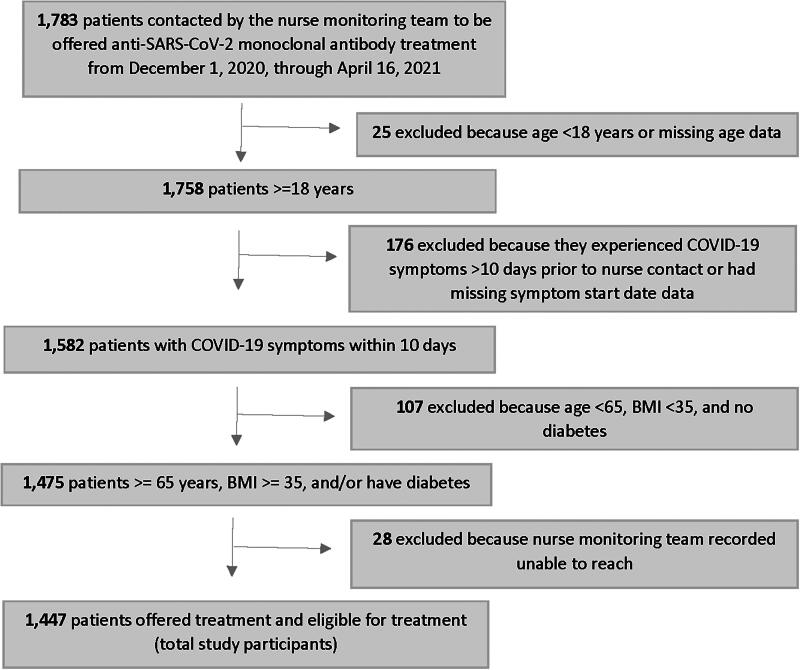
Study flowchart.

A total of 1,783 people were contacted by the nurse monitoring team to be offered anti-SARS-CoV-2 monoclonal antibody treatment from December 1, 2020, through April 16, 2021 (Fig. 1). Among them, 336 were excluded from the analysis either because these individuals were not eligible to receive anti-SARS-CoV-2 monoclonal antibody treatment under CNHS criteria or the nurse monitoring team was not able to contact them. Within the excluded group, 25 (1.4%) were under 18 years old, 176 (9.9%) people experienced symptoms of COVID-19 more than 10 days prior to the phone call or had missing symptom start date data, and 107 (6.0%) people were younger than 65, had a BMI lower than 35, and did not have diabetes. An additional 28 (1.6%) people were excluded from the analysis because the nurse monitoring team recorded that they were unable to reach those patients. This led to a final study sample of 1,447 individuals.

### Patient characteristics

Among the 1,447 participants, 813 (56.2%) were treated and 634 (43.8%) declined treatment (Table 1). Treated participants were slightly older on average than untreated participants, with a mean age of 55.7 years (SD: 16.0) compared to 50.6 years (SD: 17.8). Approximately three-fifths of both treated and untreated participants were female. BMI was also similar between treated and untreated participants, with a mean BMI of 38.2 (SD: 13.5) among treated participants compared with 38.8 (SD: 12.1) among untreated participants. A statistically significant higher proportion of treated participants had diabetes, heart disease, chronic kidney disease, and chronic lung disease compared to untreated participants.

**Table 1.** Demographic Characteristics and COVID-19-Related Outcomes of Patients Who Received and Did Not Receive Anti-SARS-CoV-2 Monoclonal Antibody Treatment for COVID-19, Cherokee Nation Health Services, December 1, 2020, to April 16, 2021

Characteristic	Treated (*n* = 813) *n* (%) or mean (SD)	Not treated (*n* = 634) *n* (%) or mean (SD)	*p* value
Covariates			
Age, mean (SD)	55.7 (16.0)	50.6 (17.8)	<0.001
Age ≥65	294 (36.2)	183 (28.9)	0.003
Sex			
Female	467 (57.4)	385 (60.7)	0.21
Male	346 (42.6)	249 (39.3)	
BMI, mean (SD)	38.2 (13.5)	38.8 (12.1)	0.37
BMI ≥35	528 (65.1)	433 (72.8)	0.002
Diabetes	414 (50.9)	239 (37.7)	<0.001
Heart disease/hypertension	566 (69.6)	212 (33.4)	<0.001
Chronic kidney disease	77 (9.5)	21 (3.3)	<0.001
Chronic lung disease	51 (6.2)	10 (1.6)	<0.001
Days between symptom onset and treatment, mean (SD)^a^	4.9 (2.2)	NA	NA
Days between symptom onset and treatment^a^	802 (100.0)	NA	NA
1–3 days	229 (28.6)	NA	NA
4–6 days	375 (46.8)	NA	NA
≥7 days	198 (24.7)	NA	NA
Outcomes			
All-cause emergency room visit	82 (10.1)	81 (12.8)	0.11
COVID-19-related emergency room visit	54 (6.6)	64 (10.1)	0.02
All-cause hospitalization	8 (1.0)	12 (1.9)	0.14
COVID-19-related hospitalization	5 (0.6)	12 (1.9)	0.03
All-cause intensive care unit visit	1 (0.1)	2 (0.3)	0.59
COVID-19-related intensive care unit visit	1 (0.1)	2 (0.3)	0.59
All-cause patient death	4 (0.5)	5 (0.8)	0.52
COVID-19-related patient death	2 (0.3)	2 (0.3)	0.99

^a^ Among patients who were treated with monoclonal antibodies and had date information available for both symptom onset and monoclonal antibody infusion (*n* = 802).

NA, not applicable; SD, standard deviation.

Among the participants in the exposure group, the average number of days between COVID-19 symptom onset and treatment was 4.9 (SD: 2.2) days, with almost a third (28.6%) of patients receiving treatment 1–3 days after symptom onset and nearly half (46.8%) receiving treatment 4–6 days after symptom onset (Table 1).

### Proportions of patients with study outcomes

The proportion of treated participants who experienced all-cause emergency room visits (10.1%) was lower compared to untreated participants (12.8%) (Table 1). The proportions of treated participants versus untreated patients who experienced COVID-19-related emergency room visits (6.6% vs. 10.1%), all-cause hospitalizations (1.0% vs. 1.9%), COVID-19-related hospitalizations (0.6% vs. 1.9%), all-cause intensive care unit visits (0.1% vs. 0.3%), COVID-19-related intensive care unit visits (0.1% vs. 0.3%), and all-cause death (0.5% vs. 0.8%) were lower. The proportions of treated participants versus untreated patients who experienced COVID-19-related death (0.3% vs. 0.3%) were the same.

### Associations between anti-SARS-CoV-2 monoclonal antibody treatment and all-cause and COVID-19-related emergency department visit, hospitalization, intensive care unit visit, and death

In bivariate analyses, there was a statistically significant difference between treated and untreated participants experiencing a COVID-19-related emergency room visit (OR, 0.63; 95% CI, 0.43–0.93) and a COVID-19-related hospitalization (OR, 0.32; 95% CI, 0.11–0.92) (Table 2). When adjusted for age, sex, BMI, diabetes, heart disease/hypertension, chronic kidney disease, and chronic lung disease, all-cause and COVID-19-related emergency room visits, and hospitalization outcomes were significantly different between groups (all-cause emergency room visit aOR, 0.50; 95% CI, 0.35–0.71; COVID-19-related emergency room visit aOR, 0.42; 95% CI, 0.27–0.63; all-cause hospitalization aOR, 0.17; 95% CI, 0.06–0.47; and COVID-19-related hospitalization aOR, 0.10; 95% CI, 0.03–0.31) (Table 2). There were no statistically significant differences in other outcomes of interest (bivariate analysis: all-cause emergency room visit OR, 0.77; 95% CI, 0.55–1.06; all-cause hospitalization OR, 0.52; 95% CI, 0.21–1.27; both all-cause and COVID-19-related intensive care unit visit OR, 0.39; 95% CI, 0.04–4.30; all-cause death OR, 0.62; 95% CI, 0.17–2.33; and COVID-19-related death OR, 0.78; 95% CI, 0.11–5.55; Adjusted analysis: both all-cause or COVID-19-related intensive care unit visits aOR, 0.06; 95% CI, 0.00–1.62; all-cause death aOR, 0.27; 95% CI, 0.05–1.17; and a COVID-19-related death aOR, 0.38; 95% CI, 0.04–3.26).

**Table 2.** Crude and Adjusted Associations Between Receipt of Anti-SARS-CoV-2 Monoclonal Antibody Treatment for COVID-19 and Adverse COVID-19-Related Outcomes, Cherokee Nation Health Services, December 1, 2020, to April 16, 2021

Outcome	Treated (*n* = 813) *n* (%)	Not treated (*n* = 634) *n* (%)	Crude odds ratio (95% CI)	Crude *p* value	Adjusted odds ratio (95% CI)^a^	Adjusted *p* value^a^
All-cause emergency room visit	82 (10.1)	81 (12.8)	0.77 (0.55–1.06)	0.11	0.50 (0.35–0.71)	<0.001
COVID-19-related emergency room visit	54 (6.6)	64 (10.1)	0.63 (0.43–0.93)	0.03	0.42 (0.27–0.63)	<0.001
All-cause hospitalization	8 (1.0)	12 (1.9)	0.52 (0.21–1.27)	0.15	0.17 (0.06–0.47)	<0.001
COVID-19-related hospitalization	5 (0.6)	12 (1.9)	0.32 (0.11–0.92)	0.03	0.10 (0.03–0.31)	<0.001
All-cause intensive care unit visit	1 (0.1)	2 (0.3)	0.39 (0.04–4.30)	0.44	0.06 (0.00–1.62)	0.18
COVID-19-related intensive care unit visit	1 (0.1)	2 (0.3)	0.39 (0.04–4.30)	0.44	0.06 (0.00–1.62)	0.18
All-cause patient death	4 (0.5)	5 (0.8)	0.62 (0.17–2.33)	0.48	0.27 (0.05–1.17)	0.08
COVID-19-related patient death	2 (0.3)^b^	2 (0.3)^b^	0.78 (0.11–5.55)	0.80	0.38 (0.04–3.26)	0.34

^a^ Adjusted for age, sex, BMI, diabetes, heart disease/hypertension, chronic kidney disease, and chronic lung disease.

^b^ Rounded to the nearest tenth place for consistency. The actual proportions of COVID-19-related patient death differ slightly for treated and not treated patients (Treated: 0.25%; Not treated: 0.32%).

BMI, body mass index; CI, confidence interval.

Among the subset of patients who received anti-SARS-CoV-2 monoclonal antibody treatment, there were no statistically significant differences in the occurrence of any study outcomes by the number of days between symptom onset and receipt of treatment in stratified analyses (Table 3).

**Table 3.** COVID-19-Related Outcomes of Patients Who Received Anti-SARS-CoV-2 Monoclonal Antibody Treatment for COVID-19, Stratified by Days Between Symptom Onset and Receipt of Monoclonal Antibody Treatment, Cherokee Nation Health Services, December 1, 2020, to April 16, 2021

Outcome, stratified by days^a^ (*N* = 802)	Outcome occurred *n* (%)	Outcome did not occur *n* (%)	Total *N*	*p* value^b^
COVID-19-related emergency room visit	54 (6.73)	748 (93.27)		0.655
≤3 days	17 (7.42)	212 (92.58)	229	—
4–6 days	22 (5.87)	353 (94.13)	375	—
≥7 days	15 (7.58)	183 (92.42)	198	—
COVID-19-related hospitalization	5 (0.62)	797 (99.38)		0.278
≤3 days	1 (0.44)	228 (99.56)	229	—
4–6 days	4 (1.07)	371 (98.93)	375	—
≥7 days	0 (0.00)	198 (100.00)	198	—
COVID-19-related intensive care unit visit	1 (0.12)	801 (99.88)		0.286
≤3 days	1 (0.44)	228 (99.56)	229	—
4–6 days	0 (0.00)	375 (100.00)	375	—
≥7 days	0 (0.00)	198 (100.00)	198	—
COVID-19-related patient death	2 (0.25)	800 (99.75)		0.578
≤3 days	0 (0.00)	229 (100.00)	229	—
4–6 days	1 (0.27)	374 (99.73)	375	—
≥7 days	1 (0.51)	197 (99.49)	198	—

^a^ Stratified by days between COVID-19 symptom onset and receipt of monoclonal antibody treatment for COVID-19.

^b^ Assessed using chi-square and Fisher’s exact tests.

## Discussion

This is the largest retrospective cohort study to assess anti-SARS-CoV-2 monoclonal antibody treatment among AI/AN people with COVID-19. In this study of 1,447 AI/AN people, anti-SARS-CoV-2 monoclonal antibody treatment was associated with 58% lower odds of experiencing a COVID-19 emergency room visit and 90% lower odds of experiencing a COVID-19-related hospitalization in adjusted analyses.

### Health equity implications

Although prior studies, including observational studies in real-world settings, have demonstrated the effectiveness of monoclonal antibody treatments for high-risk patients with COVID-19 that did not require hospitalization, these studies have not included sufficient sample sizes of AI/AN people, and most have focused on all-cause versus COVID-19-related outcomes.^25–30^ This study is the largest to specifically demonstrate that anti-SARS-CoV-2 monoclonal antibody treatment may be associated with reduced COVID-19-related emergency room visits and hospitalizations among AI/AN people in a real-world setting. Although monoclonal antibody treatments are no longer used for COVID-19 treatment, in future pandemic settings, it is probable that this class of treatments will be utilized again due to the relative speed at which they can be produced.

Intersecting historical, systemic, structural, and institutional factors results in disadvantaged social determinants of health and equity/inequity among AI/AN people. Several historical and ongoing factors have contributed to these inequities including the transgenerational effects of colonialism, racism, and trauma and significant barriers to accessing health care.^4,31,32^ Medical research must account for how disadvantaged social determinants of health and equity/inequity may result in diverse responses to medical treatments, including COVID-19 treatment, among AI/AN people compared to white people and the general population. In addition, researchers must consider the historical and ongoing mistrust of the western medical system and researchers among AI/AN people, given the system’s atrocities and injustices against AI/AN people over time.^3,5^

Cherokee Nation participates in clinical trials to support researchers in designing treatment that is safe and effective for AI/AN populations.^33^ This project is one of many of Cherokee Nation’s endeavors to support the well-being of AI/AN individuals within the reservation.^34–38^

To advance health equity, it is critical to have representation of AI/AN in clinical trials and other research. This project is an example of how community partnerships with Indian Health Service, Tribal, and Urban Indian health systems can strengthen the evidence for new and emerging treatments, address past harm, and advance equity.

## Limitations

This study has limitations. First, the landscape of monoclonal antibody treatment for COVID-19 is continuously shifting, and there are no FDA-approved monoclonal antibody treatments at this time for high-risk ambulatory patients with COVID-19. Bamlanivimab, the anti-SARS-CoV-2 monoclonal antibody treatment used in this study, although effective in treating the variant of COVID-19 that was circulating at the time, is not effective in treating the currently circulating COVID-19 variants. Although bamlanivimab is no longer used to treat the current COVID-19 variants,^39^ this study demonstrates that using anti-SARS-CoV-2 monoclonal antibody treatment effectively for the circulating variant during the study period reduced the odds of COVID-19-related emergency room visits and hospitalizations among AI/AN people. In addition, CNHS did not have the capacity to sequence the COVID-19 variants of patients in this retrospective study. It is likely that the most common circulating variant at the time was alpha, B.1.1.7, based on the Centers for Disease Control and Prevention Morbidity and Mortality Weekly Report.^18^

Second, this study is subject to potential selection bias. Participants were only eligible for study enrollment if they responded to the team contacting patients for treatment. There may be systematic differences between people who did and did not respond to the nurse monitoring team. Nevertheless, less than 2% of those who were contacted did not respond.

People who answered the phone and agreed to receive anti-SARS-CoV-2 monoclonal antibody treatment may be inherently different from those who declined. A higher proportion of those who accepted treatment experienced comorbidities than those who did not accept treatment. People experiencing these comorbidities would have been expected to experience higher odds of adverse COVID-19-related outcomes.

Nurses and medical assistants measured and recorded the weight and height of patients. Based on these measurements, the electronic health system automatically calculates the BMI and populates it in the patient medical records throughout their time as a patient at CNHS. The BMI measurement extracted for use in this study was the one recorded closest to the date the patient received anti-SARS-CoV-2 monoclonal antibody treatment or was admitted to the emergency department or hospital. The BMI of study participants who refused treatment and were not admitted to the emergency department or hospital was the last recorded BMI in their medical record. It is possible this group had outdated BMI information.

Statistical models were adjusted for potential measurable confounders; however, there may be residual confounding due to unmeasured differences between those who agreed to or declined treatment. It is likely that these baseline differences between groups attenuated the study results.

Third, the eligibility criteria of this study limit the generalizability of findings. Given the high volume of patients meeting the EUA criteria and the limited availability of treatment, CNHS had to prioritize treatments based on specific patient risk factors. As a result, findings from this study may not be generalizable to the general population of AI/AN people served through CNHS who test positive for COVID-19 with other risk factors for disease progression.

Fourth, AI/AN people may use health care systems outside of CNHS, and some patients may have obtained COVID-19 care outside of the system. Therefore, we may not know the COVID-19-related outcomes for some individuals. However, given that patients had to have received previous care through CNHS to have an existing EMR and be contacted to receive monoclonal antibody treatment, it is likely that most study participants sought care within CNHS for subsequent health outcomes.

Fifth, the sample size for this study, although larger than Close et al.’s existing study^15^ of monoclonal antibody treatment for COVID-19 among AI/AN people, was small, with fewer than 10 observations in many of the outcome groups for both treated and untreated participants. The initial intent of the study team was to use propensity score matching as a primary analysis. However, as a result of the sample size, logistic regression statistical models were a more appropriate fit (Supplementary Table S1).

## Conclusion

Anti-SARS-CoV-2 monoclonal antibody treatment was associated with lower odds of COVID-19-related emergency room visits and hospitalization in AI/AN patients at high risk for progressing to severe COVID-19.

Future studies that prospectively assess COVID-19-related outcomes after monoclonal antibody treatment across multiple AI/AN communities and community members, including those who are not at high risk for progressing to severe COVID-19, would be helpful for understanding whether these findings can be generalized more broadly across AI/AN communities. Future studies should include AI/AN people when evaluating novel treatments for COVID-19 and other conditions/diseases to advance health equity.

## Abbreviations Used

AI/AN: American Indian and Alaska Native
AOR: Adjusted odds ratio
BMI: Body mass index
CI: Confidence interval
CNHS: Cherokee Nation Health Services
COVID-19: Coronavirus disease 2019
EMR: Electronic medical record
EUA: Emergency use authorization
FDA: Food and Drug Administration
OR: Odds ratio
PCR: Polymerase chain reaction
SARS-CoV-2: Severe acute respiratory syndrome coronavirus 2
SD: Standard deviation
STROBE: Strengthening the Reporting of Observational Studies in Epidemiology
