# Supplementary material for: Advancing Health Equity for American Indian and Alaska Native People Through Inclusion in Clinical Trials: Anti-SARS-CoV-2 Monoclonal Antibody Treatment and COVID-19 Outcomes Among Ambulatory Cherokee Nation Health Services Patients
Source: Health Equity. 2025 Apr 21;9(1):235–44. doi: 10.1089/heq.2024.0185 (PMC12270532; doi:10.1089/heq.2024.0185)
Supplement: Supplementary Table S1 [file heq.2024.0185_supplementary_table_s1.doc]

**Supplementary Table 1. Demographic characteristics and COVID-19 related outcomes of propensity score-matched patients who received and did not receive anti-SARS-CoV-2 monoclonal antibody treatment** for COVID-19.

| **Characteristic/Covariates** | **Treated**  **(n = 595)**  **n (%) or mean (SD)** | **Not Treated**  **(n = 595)**  **n (%) or mean (SD)** | **P Value** |
| --- | --- | --- | --- |
| Age, mean (SD) | 59.18 (14.5) | 49.59 (17.8) | <.001 |
| Age over 65 | 248 (41.7) | 155 (26.1) | <.0001 |
| Sex |  |  |  |
| Female | 327 (55.0) | 362 (60.8) | 0.04 |
| Male | 268 (45.0) | 233 (39.2) |
| BMI, mean (SD) | 37.83 (13.7) | 38.84 (12.1) | 0.18 |
| BMI over 35 | 371 (62.4) | 433 (72.8) | <.0001 |
| Diabetes | 366 (6.5) | 223 (37.5) | <.0001 |
| Heart disease/ hypertension | 562 (94.5) | 199 (33.5) | <.0001 |
| Chronic kidney disease | 75 (12.6) | 20 (3.4) | <.0001 |
| Chronic lung disease | 51 (8.6) | 9 (1.5) | <.0001 |

Abbreviations: SD, standard deviation
